# Abdominal eggshell calcifications in the newborn: meconium peritonitis

**DOI:** 10.11604/pamj.2018.31.84.16592

**Published:** 2018-10-04

**Authors:** Ipek Guney Varal, Pelin Dogan

**Affiliations:** 1 Department of Pediatrics, Division of Neonatology, University of Health Sciences, Bursa Yüksek Ihtisas Teaching Hospital, Bursa, Turkey

**Keywords:** Meconium peritonitis, eggshell calcifications, newborn

## Image in medicine

**Figure 1:**
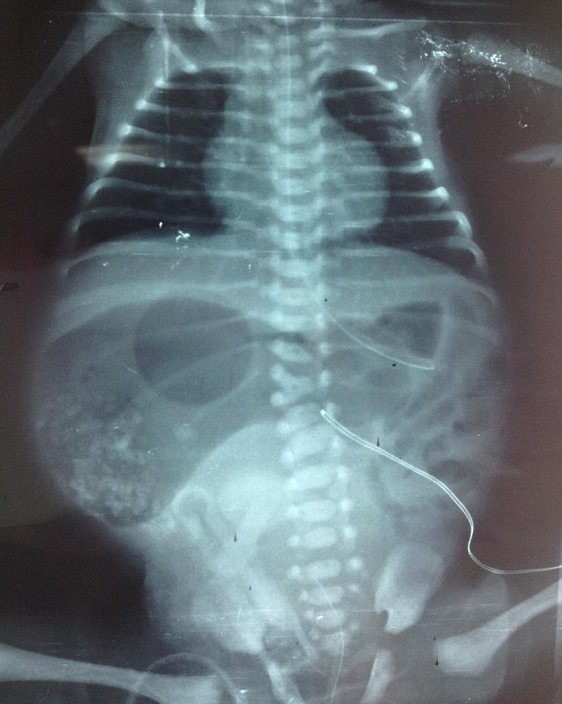
Meconium peritonitis-eggshell calcification

Meconium peritonitis is a sterile chemical peritonitis caused by intestinal perforation in-utero or shortly after birth and perforation usually occurs due to intrauterine obstruction. Its incidence is approximately 1 in 35,000 births and the mortality was reported to be 60-80%. The radiographic findings of meconium peritonitis are pneumoperitoneum, intestinal obstruction and abdominal calcifications. The classic eggshell calcification occurs owing to the defensive mechanism of the body against the inflammation in an effort to surround the meconium in the abdominal cavity. In a newborn with abdominal distension, meconium peritonitis should be kept in mind, especially if calcified foci are seen on plain abdominal radiographs, since timely diagnosis and surgery improves the survival rates. We present the case of a female newborn of 34 weeks' gestational age was admitted to the neonatal intensive care unit (NICU) with marked abdominal distension. The pregnancy was complicated by maternal polyhydramnios and a cesarean section was performed due to fetal distress. The infant was noted to have a grossly distended abdomen; however, the physical examination was otherwise unremarkable. The abdominal radiograph revealed distension of the bowel loops and free air with multiple peritoneal calcifications (Figure 1). Surgery, performed on day 1 of life, demonstrated ileal atresia and perforation in the distal part of the ileum. Solid meconium, which leaked into the abdominal cavity was removed and the atresic bowel was resected and anastomosed. The infant regained bowel function on postoperative day 7 and discharged home on the third week after gradual advancement to full enteral feeding.

